# Medical College Notes

**Published:** 1895-06

**Authors:** 


### Medical College Notes.

THE University of Buffalo has added a new department—namely, the department of pedagogy. A faculty has already been chosen in part, and Mr. B. B. Glenny made a member of the council of the university to represent this department. Lectures will begin in September, and it may be confidently predicted that this much-needed school will prove a success. It will be modeled after the best schools of pedagogy in this country.

---

THE Albany Medical College graduated a class of forty-seven April 16, 1895. The alumni association held its twenty-second annual reunion. The report of the recording secretary shows the alumni roll to be 1,243; total number of graduates, 2,055. The following were elected as officers: Shobald Smith, '83, of Washington, president; corresponding secretary, J. B. Stonehouse, '75. —*Medical Standard.*

